# The Fine Line Between Chaos and Creation: Exploring the Link Between Borderline Personality Disorder Traits and Creativity

**DOI:** 10.1192/j.eurpsy.2025.1923

**Published:** 2025-08-26

**Authors:** M. Rosa, T. Rocha, J. F. Cunha, J. Moura, J. Leal, I. Lopes, D. Seabra, N. Ramalho

**Affiliations:** 1 DPSM, ULS Arco Ribeirinho, Barreiro, Portugal

## Abstract

**Introduction:**

Borderline Personality Disorder (BPD) is primarily characterized by emotional dysregulation, impulsivity, and unstable relationships. While much of the research on BPD focuses on its impairments, recent studies suggest a possible link between certain BPD traits and enhanced creativity. Emotional intensity, cognitive disinhibition, and non-linear thinking—common in individuals with BPD—are also characteristics frequently associated with creative processes. This review aims to synthesize existing literature exploring the connection between BPD traits and creativity.

**Objectives:**

The primary objective is to review and summarize the existing literature on the association between BPD traits and creativity, focusing on how emotional and cognitive aspects of BPD may facilitate creative thinking.

**Methods:**

A non-systematic review of the literature was conducted by searching academic databases such as PubMed, PsycINFO, and Google Scholar for articles published up to 2023. Search terms included “Borderline Personality Disorder,” “creativity,” “personality traits,” and “emotion regulation.” Articles were selected based on relevance to the topic, focusing on studies that explored the link between BPD traits and creativity.

**Results:**

The review identified studies showing a nuanced relationship between BPD traits and creativity. Individuals with BPD traits may access a wider range of emotional experiences, which can enhance creative expression. Impulsivity was linked to spontaneous idea generation and creative risk-taking, although it may also hinder the focus needed for refining creative work. Some research points out that the extreme emotional dysregulation associated with BPD can undermine creative productivity. While emotional intensity might inspire originality, it can also lead to difficulty in structuring ideas, or an inability to complete creative tasks. A few studies even suggest that high levels of interpersonal difficulties, common in individuals with BPD, may further complicate the consistent pursuit of creative projects, as social conflicts or emotional crises might divert focus and energy. In contrast, a smaller body of literature found that individuals with BPD traits might excel in creative fields that value expression, such as art, music, or writing, where emotional intensity and non-linear thinking can be channeled into meaningful creative outputs.

**Conclusions:**

While the reviewed literature suggests that certain BPD traits may enhance creativity, the relationship is complex and multifaceted. Emotional intensity and impulsivity, while potentially beneficial for creative output, can also disrupt focus and long-term creative endeavors. Overall, while BPD traits may foster creativity, they also pose challenges to sustaining long-term creative productivity.

**Disclosure of Interest:**

None Declared

